# Evaluation of an articulated passive ankle–foot prosthesis

**DOI:** 10.1186/s12938-022-00997-6

**Published:** 2022-04-27

**Authors:** Elke Lathouwers, Toon Ampe, María Alejandra Díaz, Romain Meeusen, Kevin De Pauw

**Affiliations:** 1 Human Physiology and Sports Physiotherapy Research Group, Vrije Universiteit Brussel, 1050 Brussels, Belgium; 2 Brussels Human Robotics Research Center (BruBotics), Vrije Universiteit Brussel, 1050 Brussels, Belgium

**Keywords:** Lower-limb amputation, Ankle, Foot prosthesis, Performance, Functional mobility

## Abstract

**Background:**

Current ankle prostheses for people with unilateral transtibial amputation (TTA) or transfemoral amputation (TFA) are unable to mimic able-bodied performance during daily activities. A new mechanical ankle–foot prosthesis was developed to further optimise the gait of people with a lower-limb amputation. This study aimed to evaluate the Talaris Demonstrator (TD) during daily activities by means of performance-related, physiological and subjective outcome measures.

**Materials and methods:**

Forty-two participants completed a protocol assessing performance and functional mobility with their current prosthesis and the TD. The protocol comprised the L-test, 2 min of stair climbing, 2 min of inclined treadmill walking, 6 min of treadmill walking at 3 different speeds in consecutive blocks of 2 min, and a 3-m Backward Walk test (3mBWT). Heart rate was measured during each task, and oxygen uptake was collected during all tasks except for the L-test and 3mBWT. Time of execution was recorded on the L-test and 3mBWT, and the rate of perceived exertion (score = 6–20), fatigue and comfort (score = 0–100) were assessed after each task. Paired sample t-tests and Wilcoxon Signed-rank tests were performed to compare outcomes between prosthetic devices. Benjamini–Hochberg corrections were applied to control for multiple comparisons with a level of significance set at *α* = 0.05.

**Results:**

Subjects with a TTA (*N* = 28) were faster with their current prosthesis compared to the TD on the L-test and 3mBWT (*p* = 0.005). In participants with a TFA (*N* = 14), we observed a tendency towards a higher heart rate during the L-test and towards increased comfort during inclined walking, with the TD compared to the participants’ current prosthetic device (0.05 < *p* < 0.10). Further, no significant results were observed.

**Conclusion:**

The Talaris Demonstrator is a novel state-of-the-art passive ankle–foot prosthesis for both people with a TTA and TFA. Subjective measures indicate the added value of this device, while overall task performance and intensity of effort do not differ between the Talaris Demonstrator and the current prosthesis. Further investigations unravelling both acute and more prolonged adaptations will be conducted to evaluate the TD more thoroughly.

## Introduction

After lower limb amputation, individuals with a transfemoral amputation (TFA) and transtibial amputation (TTA) require an ankle–foot prosthesis to regain their ability to ambulate [1]. Nowadays, three different types of ankle–foot prostheses are marketed, namely passive, quasi-passive, and active devices [2, 3]. Passive prostheses rely on a fixed spring and offer basic functionality [4]. Passive ankle–foot prostheses are most commonly used as they are inexpensive and implement cushioning during gait [5]. Unfortunately, those passive ankle–foot prostheses are still unable to mimic able-bodied performance during daily activities as they fail to provide sufficient range of motion and net positive joint work [6, 7]. This induces higher metabolic energy consumption during daily activities, asymmetrical limb loading, and altered gait patterns compared to able-bodied individuals [8–10]. Quasi-passive prostheses are also mechanical but equipped with microprocessor technology enabling them to adapt to situations such as walking on an inclined surface [4]. They are incapable of generating power but store and release energy to support forward propulsion. Active or powered prostheses can generate an external force through an actuator and therefore have the ability to mimic intact limb functional characteristics [2, 11].

Mimicking the human ankle function is one of the biggest challenges in replicating non-pathological gait due to the loss of function reducing ankle push-off power during walking [7, 12]. Despite the transition from passive to quasi-passive and more recently to active prosthetic devices, which try to alleviate the loss of function by means of an actuator, the challenge remains [13]. An explanation for not attaining able-bodied functioning is the limited range of motion of the conventional prosthetic ankle joint [14, 15]. The absence of an articulated ankle joint entails increased compensatory strategies such as increased muscle activity of the intact limb and trunk, increased intact limb loading, damping, and increased trunk rotation [16, 17]. Subsequently, these compensations cause balance disturbance and lead towards an increased risk of falling, which in turn lowers the quality of life [18–20]. Furthermore, reduced mobility and gait asymmetries contribute to the increased onset of low back pain, arthritis of the healthy knee, bilateral hip osteoarthritis, reduced hip bone density of the amputated limb, and muscle atrophy in people with a unilateral lower-limb amputation compared to able-bodied individuals [21–24]. Novel prosthetic devices strive towards restoring physical functioning and preventing secondary injuries by mimicking the gait pattern of able-bodied individuals as closely as possible by obtaining gait symmetry and optimizing comfort. Reducing postural imbalances, enhancing prosthetic functioning, and thereby lowering the onset of secondary injures and thus improving quality of life requires research into the development of ankle–foot prostheses with an articulated ankle joint [25–27]. Therefore, a new passive ankle–foot prosthesis (i.e., Talaris Demonstrator) was developed.

The Talaris Demonstrator (TD) is based on previous research with the Ankle Mimicking Prosthetic (AMP-) Foot, with the fundamental aim of developing a new prosthetic ankle that restores normal gait and posture of individuals with lower-limb amputation [28–31]. The TD is a prototype in the development process of an innovative passive ankle prosthesis classified as energy storing and releasing foot. The prosthetic ankle is dorsiflexed during the stance phase while a plantar-flexion torque is applied to the ankle. This plantar-flexion torque is provided by the passive elastic components, supporting the user's forward movement while walking. When leaning forward on the intact limb at the end of the stance phase, the elastic component returns to its initial position and produces a passive supporting push-off. The novelty of the foot compared to most prostheses currently being used by people with an amputation is the articulating ankle joint. This articulating joint allows for plantar and dorsal flexion of the foot and adaptivity to changing environmental conditions enhancing user comfort and safety.

Among daily activities, walking is an essential and undeniable factor influencing quality of life in people with a lower limb amputation [32]. Consequently, the assessment of functional performance is usually conducted through gait tasks in which physiological, biomechanical and subjective information is often collected (e.g., 6 min of walking, stair climbing, and incline walking) to evaluate an individual’s functional capacity, gait ability, balance and postural control [33–36]. To assess the functionality of this prototype and its impact on an individual with lower limb amputation, the purpose of this study is to evaluate the TD during daily activities by means of performance-related, physiological, and subjective outcome measures.

## Results

### Participants’ characteristics

In total, 42 participants completed the study protocol, whereof 28 with a unilateral TTA (female = 5, male = 23) and 14 with a unilateral TFA (female = 3, male = 11). Among individuals with a TTA (right-sided amputation = 8), limb loss was caused by trauma (*n* = 19), vascular problems (*n* = 5) and cancer (*n* = 4). Reasons for limb loss within participants with a TFA (right-sided amputation = 6) were trauma (*n* = 8), cancer (*n* = 5) and congenital (*n* = 1). Furthermore, within individuals with a TTA, 27 had a passive ankle–foot prosthesis and 1 had a quasi-passive one. Within individuals with a TFA, these numbers were 13 and 1, respectively. Participants’ characteristics are displayed in Table 1 and details regarding the individuals' current prosthesis are provided in Table 3 in Appendix. Additionally, the EuroQol-5D questionnaire revealed that the individuals with a TTA indicated having no problems, slight and moderate problems for mobility (*n* = 22, *n* = 5 and *n* = 1, respectively) and pain (*n* = 13, *n* = 13 and *n* = 2, respectively). Furthermore, they reported having slight and moderate problems with anxiety (*n* = 27 and *n* = 1, respectively), having no problems, slight and severe problems with daily activities (*n* = 22, *n* = 5 and *n* = 1, respectively) and having no problems with selfcare. Participants with a TFA reported no problems and slight problems with mobility (*n* = 9 and *n* = 5, respectively), selfcare (*n* = 13 and *n* = 1, respectively), daily activities (*n* = 10 and *n* = 4, respectively) and pain (*n* = 7 and *n* = 7, respectively), whereas they reported having no problems with anxiety. In terms of perceived health, no significant difference was detected (*p* = 0.885) between participants with a TTA (86.2 ± 9.4) and TFA (85.9 ± 6.3).

**Table 1:** Overview of participants' characteristics displayed as mean ± standard deviation

	Participants with TTA (*n* = 28)	Participants with TFA (*n* = 14)
Age (years)	49.2 ± 12.3	46.1 ± 14.9
Weight (kg)	80.4 ± 11.8	80.8 ± 12.3
Height (cm)	176.7 ± 6.9	175.6 ± 7.6
Residual limb length (cm)	16.7 ± 4.9	36.0 ± 8.5
Time since amputation (years)	12.8 ± 12.0	15.0 ± 13.4
Oxygen consumption at rest (ml · (kg · min)^−1^)	7.8 ± 1.5	7.6 ± 1.4
Heart rate at rest (bpm)	86 ± 15	82 ± 13
Visual analogue scale comfort at rest	79 ± 22	86 ± 15
Visual analogue scale fatigue at rest	21 ± 25	23 ± 31

### Performance and functional mobility

Table 2 details the means, standard deviations and p-values of the tasks performed with the TD and the current prosthesis. However, within this paragraph the means and standard deviations refer to the pooled means and standard deviations of both prosthetic devices.

**Table 2:** Results assessment of performance and functional mobility

			Time (s)	Speed (m s^−1^)	Heart rate (bpm)	Oxygen consumption [ml · (kg · min)^−1^]	VAS comfort	VAS fatigue	RPE
L-test	TTA	Current	18.38 ± 3.47	/	102 ± 12	/	77 ± 25	18 ± 21	9 ± 2
		TD	19.66 ± 3.71	/	100 ± 13	/	74 ± 24	21 ± 23	9 ± 2
		*p*-value	**0.005**	/	0.313	/	0.401	0.429	0.441
	TFA	Current	24.62 ± 5.80	/	98 ± 13	/	79 ± 18	30 ± 30	9 ± 2
		TD	25.81 ± 7.72	/	101 ± 12	/	80 ± 14	26 ± 28	9 ± 2
		*p*-value	0.200	/	0.068	/	0.439	0.414	0.323
Stair climbing	TTA	Current		60 ± 16*	141 ± 14	24.1 ± 6.5	63 ± 18	58 ± 24	14 ± 2
		TD		59 ± 17*	140 ± 14	23.5 ± 6.1	65 ± 20	54 ± 22	13 ± 2
		*p*-value		0.401	0.488	0.401	0.401	0.401	0.330
	TFA	Current	/	/	/	/	/	/	/
		TD	/	/	/	/	/	/	/
		*p*-value	/	/	/	/	/	/	/
Slope walking	TTA	Current	/	3.2 ± 0.9	122 ± 16	15.8 ± 4.4	68 ± 27	36 ± 20	12 ± 2
		TD	/	3.2 ± 0.8	123 ± 14	16.5 ± 5.1	75 ± 21	28 ± 18	11 ± 2
		*p*-value	/	0.401	0.412	0.401	0.401	0.084	0.401
	TFA	Current	/	2.8 ± 0.9	111 ± 15	14.6 ± 2.9	71 ± 21	39 ± 30	10 ± 3
		TD	/	3.0 ± 1.2	115 ± 15	15.0 ± 4.3	85 ± 12	31 ± 29	10 ± 3
		*p*-value	/	0.170	0.206	0.349	0.068	0.170	0.448
6MWT	TTA	Current	/	SS: 3.5 ± 0.9 75% SS: 2.6 ± 0.7 125% SS: 4.4 ± 1.1	SS: 108 ± 15 75% SS: 104 ± 14 125% SS: 113 ± 17	SS: 11.8 ± 1.8 75% SS: 10.6 ± 2.0 125% SS: 13.4 ± 3.3	71 ± 26	32 ± 22	SS: 10 ± 2 75% SS: 9 ± 3 125% SS: 11 ± 2
		TD	/	SS: 3.3 ± 0.7 75% SS: 2.5 ± 0.5 125% SS: 4.2 ± 0.8	SS: 109 ± 14 75% SS: 105 ± 16 125% SS: 111 ± 15	SS: 12.0 ± 2.5 75% SS: 11.1 ± 2.8 125% SS: 13.6 ± 3.4	75 ± 20	35 ± 24	SS: 10 ± 2 75% SS: 9 ± 3 125% SS: 11 ± 2
		*p*-value	/	SS: 0.401 75% SS: 0.401 125% SS: 0.401	SS: 0.401 75% SS: 0.401 125% SS: 0.495	SS: 0.401 75% SS: 0.401 125% SS: 0.401	0.401	0.401	SS: 0.494 75% SS: 0.429 125% SS: 0.401
	TFA	Current	/	SS: 2.7 ± 0.8 75% SS: 2.1 ± 0.6 125% SS: 3.4 ± 1.0	SS: 103 ± 14 75% SS: 98 ± 14 125% SS: 109 ± 18	SS: 11.1 ± 2.5 75% SS: 2.1 ± 0.6 125% SS: 12.4 ± 2.7	70 ± 21	35 ± 30	SS: 9 ± 3 75% SS: 9 ± 3 125% SS: 11 ± 4
		TD	/	SS: 2.9 ± 0.9 75% SS: 2.2 ± 0.7 125% SS: 3.6 ± 1.1	SS: 105 ± 15 75% SS: 100 ± 15 125% SS: 113 ± 18	SS: 12.2 ± 2.8 75% SS: 11.5 ± 3.2 125% SS: 14.1 ± 4.5	80 ± 15	44 ± 29	SS: 9 ± 2 75% SS: 9 ± 3 125% SS: 11 ± 3
		*p*-value	/	SS: 0.184 75% SS: 0.192 125% SS: 0.192	SS: 0.112 75% SS: 0.121 125% SS: 0.154	SS: 0.112 75% SS: 0.329 125% SS: 0.112	0.112	0.121	SS: 0.349 75% SS: 0.352 125% SS: 0.121
3mBWT	TTA	Current	4.42 ± 1.36	/	106 ± 16	/	62 ± 28	20 ± 20	9 ± 2
		TD	4.98 ± 1.48	/	105 ± 15	/	73 ± 23	21 ± 22	9 ± 2
		*p*-value	**0.005**	/	0.429	/	0.200	0.429	0.317
	TFA	Current	7.04 ± 3.32	/	97 ± 12	/	72 ± 21	24 ± 28	8 ± 2
		TD	7.27 ± 3.73	/	100 ± 12	/	80 ± 21	24 ± 28	8 ± 3
		*p-*value	0.349	/	0.112	/	0.117	0.329	0.414

Mean ± SD (unless mentioned otherwise)

^*^ (spm): steps per minute; /: not applicable

VAS: visual analogue scale; RPE: rating of perceived exertion; Stair climbing: 2 min of stair climbing; Slope walking: 2 min of inclined treadmill walking at 10%; 3mBWT: 3-m Backward Walk test; 6MWT: 6 min of treadmill walking at 3 different speeds in consecutive blocks of 2 min; Current: participants’ current prosthesis; TD: Talaris Demonstrator; SS: self-selected speed; TTA: participant with a unilateral transtibial amputation; TFA: participant with a unilateral transfemoral amputation

Participants with a TTA performed the L-test and 3-m Backward Walk test (3mBWT) faster with their current prosthesis compared to the TD with mean differences of 1.28 s and 0.56 s, respectively. Heart rate during the L-test and 3mBWT did not differ between prosthetic conditions and amounted to 101 ± 13 bpm during the L-test and 105 ± 15 bpm during the 3mBWT. During stair climbing, slope walking and 6MWT, we observed no differences in walking speed (60 ± 16 steps per minute, 3.2 ± 0.8 m s^−1^, and 3.4 ± 0.8 m s^−1^, respectively), heart rate (141 ± 14 bpm, 123 ± 15 bpm and 109 ± 14 bpm, respectively) and oxygen consumption (23.6 ± 6.3 ml · (kg · min)^−1^, 16.1 ± 4.7 ml · (kg · min)^−1^ and 11.9 ± 1.2 ml (kg min)^−1^). On all five tests, we also detected no differences in terms of comfort, fatigue and perceived exertion between the two prostheses.

In participants with a TFA, we found no differences in time required to conduct the L-test (mean time 25.22 ± 6.73 s) and 3mBWT (7.15 ± 3.46 s) between the two prostheses. Heart rate during the L-test and 3mBWT did not differ either, amounting to a mean of 100 ± 13 bpm during the L-test and 98 ± 12 bpm during the 3mBWT. Nonetheless, we observed a tendency towards a higher heart rate during the L-test when walking with the TD. Participants with a TFA did not conduct 2 min of stair climbing because of safety reasons. Furthermore, we detected no differences regarding heart rate (113 ± 15 bpm and 104 ± 14 bpm, respectively), oxygen consumption (14.8 ± 3.6 ml · (kg · min)^−1^ and 11.6 ± 2.4 ml · (kg · min)^−1^, respectively) and walking speed (2.9 ± 1.0 m s^−1^ and 2.8 ± 0.8 m s^−1^, respectively) during slope walking and 6 min of treadmill walking. During the performance of the four tests, we found no differences in terms of comfort, fatigue and perceived exertion between the two prostheses. However, it must be stressed that we observed a tendency towards significance in terms of comfort during slope walking favouring the TD (mean difference = 14).

### System usability

The system usability score is a questionnaire (score = 0–100, mean = 68) that comprises 10 questions aimed at assessing the usability of the TD. Questions 2, 5 and 9 were omitted from the questionnaire because of their irrelevance. We found no difference in scores between participants with a TFA and TTA. The values were 82 ± 16 and 82 ± 17, respectively (*p* = 0.472).

## Discussion

This study aimed at evaluating the TD during daily activities by means of performance-related, physiological, and subjective outcome measures in accordance with the recently published guidelines on prosthetic evaluation [37]. Our results indicated that participants with a TTA were faster with their current prosthesis compared to the TD on the L-test and 3mBWT. Furthermore, our results revealed no significant differences regarding heart rate, oxygen consumption, comfort, perceived exertion, and fatigue in people with a TTA. Yet, we detected a tendency towards a significantly higher level of fatigue during slope walking with the participants’ current device compared to the TD. In addition, despite no differences in comfort being found, it must be noted that comfort was frequently rated higher with the TD compared to the participants’ current device. This could be explained by the more natural feel of the TD's roll-over due to its design. In participants with a TFA, we observed a tendency towards a higher heart rate during the L-test and increased comfort during slope walking and 6 min walking with the TD compared to the participants’ current prosthetic device.

De Pauw et al. found that people with a TTA during treadmill level walking had a self-selected walking speed of 4.1 ± 1.2 km/h, a slow speed of 3.1 ± 0.9 km h^−1^ and a fast speed of 5.1 ± 1.5 km h^−1^ [32]. In people with a TFA these values were 2.8 ± 0.9 km h^−1^, 2.2 ± 0.7 km h^−1^ and 3.5 ± 1.2 km h^−1^ respectively [32]. The walking speeds in our study remain within the lower bound of the standard deviations of the values of De Pauw et al. and therefore can be assumed similar. Regarding oxygen consumption, heart rate and rating of perceived exertion, the same conclusion can be drawn [32]. To the best of our knowledge there are currently no studies available on walking speed, heart rate, and oxygen consumption during 2 min slope walking and 2 min of stair climbing. Furthermore, the reference values for performing the L-test amount 29.5 ± 12.8 s in people with a TTA and 41.7 ± 16.8 s in people with a TFA [38]. In our studies people with a TTA and TFA were faster (mean difference: ± 10.5 and 16.7 s, respectively).

The significant difference between the current prosthesis and the TD in time required to complete the L-test and 3mBWT is not deemed clinically relevant as the minimal detectable change for the L-test varies between 2.15 and 3.19 s depending on the cause of amputation and as the minimal detectable change for the 3mBWT ranges between 1.52 and 2.94 s depending on the population [33, 39–41]. However, it should be noted that 3mBWT has not yet been validated for people with a limb amputation. The test has been validated originally to identify older adults at risk of falling [42]. It was found that older adults who completed the test in less than 3 s were unlikely to report falling, while those who needed more than 4.50 s were most likely to report falling. Since both older adults and people with a lower limb amputation share a high risk of falling, we opted to implement this test in our protocol [18]. We found that people with a TTA needed on average 4.70 ± 1.44 s to complete the 3mBWT and people with a TFA needed 7.15 ± 3.47 s. Further research validating this test is required to contextualise our results.

Our results generally revealed no discernible differences between the participants' current prosthesis and the TD. This can be attributed to the fact that participants were not provided with a one-month familiarization period to get accustomed to the TD [43]. Only one prototype was built for testing for financial and practical reasons. As such, it was impossible to provide participants with a prosthesis to try out over several weeks. They were allowed approximately 1 h to get acquainted with the prototype, yet this is insufficient to detect true differences in performance [43]. Nevertheless, we observed a tendency towards an instantaneously increment in comfort and decrease in fatigue while walking with the TD compared to the current prosthetic device. This implies that participants might benefit from the TD due to its articulated ankle joint providing flexibility. Furthermore, on average, both participants with a TTA a TFA scored the usability of the TD at 82 out of 100. This score indicates that the device, despite no familiarisation, is rated above average in terms of user convenience and that there is a high probability that participants will recommend the device to other peers [44].

Our study had several limitations. We faced a lot of missing data due to the differences in accommodation among the test facilities. This resulted in 2 participants with TFA being unable to perform the 2-min incline walking, 10 participants with TTA being unable to perform the 2-min stair climbing, and 3 participants with TTA unable to perform 2 min of slope walking. However, the missing data does not outweigh the number of patients we could reach by performing tests in this manner, as finding participants within this population is a challenging task. In addition, we encountered practical issues with the oxygen measuring device, as there was a mismatch between the different user pieces. According to respiratory ventilation (L min^−1^), the VO2 master is equipped with three different sizes of user pieces. Size R can measure values between 3 and 50 L min^−1^, size M between 15 and 180 L min^−1^, and size L between 25 and 250 L min^−1^. During a strenuous physical task such as climbing stairs, some participants' oxygen consumption was too high for mouthpiece R, and hence no data could be rendered. A combined R and M mouthpiece could solve this bottleneck, as the instrument is easy to handle, portable, reasonably accurate, and features a brief calibration period [45]. Table 4 in Appendix summarises the number of cases analysed per variable.

Secondly, based on the current results of this study, it might have been better to make a sample size calculation to allow for a non-inferiority analysis rather than an analysis aiming at pointing out a difference. This would have enabled us to make a statement about whether the TD is rated equally well as the individuals' current prosthesis. For this study, we estimated the required sample size based on a standard medium effect size of *d* = 0.5 as studies reporting effect sizes on functional tests evaluating different prostheses are scarce. A total of 28 participants were required to achieve a power of 80% with a level of significance set at 0.05. We did not report any effect size because we found any clinically meaningful differences between the individuals’ current prosthesis and the TD.

A final limitation is that the spring of the TD device was not tailored to the participant’s body mass nor size due to time constraints and the design of the prototype itself, possibly affecting performance, fatigue, and comfort.

## Conclusion

This study contributes to a possible beneficial effect of the passive TD for participants with a lower limb amputation due to its flexibility as a tendency towards an instantaneously increment in comfort and decrease in fatigue was found during slope walking compared to the current prosthetic device. Future scope, including biomechanical measures, allowing adequate familiarisation periods and unravelling both acute and more prolonged adaptations, needs to be conducted to evaluate the TD more thoroughly.

## Methods

Participants with a unilateral transfemoral and transtibial amputation (TFA and TTA, respectively) were recruited through contacting rehabilitation centres and orthopaedic departments of hospitals in Belgium, and through social media between March and August 2021. All participants (aged 25–75 years) completed their rehabilitation and had a Medicare Functional Classification level K2-4. Adults with a bilateral, a trans-articular knee or hip, or additional upper limb amputation, were excluded as well as participants with neurological disorders or with stump pains and wounds. All participants provided their written consent after being written and verbally informed regarding the study protocol. The study was executed in compliance with the Declaration of Helsinki [46] and was approved by the medical Ethics Committee of the University Hospital of the Vrije Universiteit Brussel (B.U.N. 143201526629) and by the Federal Agencies for Medicines and Health Products (FAGG/80M0860).

Participants visited one of the test sites according to their residence: Axiles Bionics (Brussels), Revarte (Antwerp), University Hospital (UGhent) or Matton Orthopaedics (Bruges or Ghent). On arrival, an anamnesis was performed, biometric measurements were collected and the EuroQol-5D questionnaire, measuring quality of life, was completed [47, 48]. The participants completed an experimental protocol assessing performance and functional mobility. It comprised the L-test (ICC > 0.96) [38] followed by 2 min of stair climbing [49], 2 min of inclined treadmill walking at 10% [49], 6 min of treadmill walking in consecutive blocks of 2 min and at last, a 3-m Backward Walk test (3mBWT, ICC > 0.94) [39]. Both the L-test and 3mBWT were performed 3 times. Between different trials of the L-test and 3mBWT, a 30-s pause was provided and between each task a 60-s pause was included. Regarding the consecutive blocks of 2 min during the 6 min treadmill walking, those consisted of walking at self-selected (SS) speed, at 75% of the SS speed and at 125% of the SS speed [3]. The protocol was completed with both the individuals’ current prosthesis and the TD, in a randomized order, to enable comparison. Both devices were fitted to the individuals’ preference of which the TD was fitted by adjusting the length of the pylon and pyramid connector and by customizing the amount of rigidity. After completing the protocol with the TD, the user-friendliness of the device was questioned by means of the system usability scale [50].

Oxygen uptake (VO_2_, ml·kg^−1^·min^−1^) was measured by means of a portable ergospirometer (VM Pro, VO_2_ Master Health Sensors Inc., Canada) (typical error for absolute VO_2_ < 0.3L·min^−1^) [45]. Data was transmitted in real-time to the VO2master’s mobile application (VO2 Master Manager). Heart rate (beats per minute) was measured continuously by means of a chest strap (Cyclus2, RBM elektronik-automation GmbH, Germany) and was also transmitted in real-time to the same application. The VM Pro Analyzer was calibrated according to the manufacturer’s guidelines before each test after which baseline measurements were collected (i.e. heart rate, oxygen uptake, and level of comfort and fatigue by means of visual analogue scales (VAS comfort & VAS fatigue, respectively)) [51]. Heart rate was measured during each task and oxygen uptake was collected on all tasks except for the L-test and 3mBWT since these two tests are too short to obtain reliable data on oxygen uptake [52]. Time was recorded on the L-test and 3mBWT, and the rating of perceived exertion [53], fatigue and comfort were assessed after each task.

### Data processing and statistical analysis

The collected data were imported to excel. On all tasks excluding the L-test and 3mBWT, heart rate data and oxygen consumption were averaged over de last 10 s of the measurements. Once data was processed, the excel file was imported in IBM SPSS Statistics, version 27 (IBM Corp., Armonk, N.Y., USA) for statistical analysis. Shapiro–Wilk tests were applied to access normality. If normality was assumed, a paired sample t-test was applied. If normality was not assumed, the non-parametric Wilcoxon Signed-rank test was performed. Benjamini–Hochberg corrections were applied to control for multiple comparisons with a level of significance set at *α* = 0.05.

## Data Availability

The authors declare that all data supporting the findings of this study are available within this article.

## Appendix

See Tables 3 and 4.

**Table 3:** Individuals' current prosthesis

Participant	Level of amputation	Ankle–foot prosthesis	Knee prosthesis
1	TFA	Flex-Foot Assure®	OTTOBOCK 3R106
2	TTA	OTTOBOCK Trias	
3	TTA	Pro-Flex® LP	
4	TTA	RUSH Foot HiPro	
5	TTA	High profile pro-flex®	
6	TFA	Freedom Highlander®	Freedom Plié® 3
7	TTA	OTTOBOCK Triton	
8	TTA	OTTOBOCK Taleo Harmony	
9	TTA	Proprio Foot®	
10	TTA	OTTOBOCK Triton HD	
11	TFA	OTTOBOCK Trias	OTTOBOCK Genium
12	TTA	Pro-Flex Pivot®	
13	TTA	Re-Flex Shock TM	
14	TTA	Pro-Flex® XC	
15	TTA	Vari-Flex®	
16	TFA	OTTOBOCK Taleo LP	OTTOBOCK Genium
17	TTA	Pro-Flex® XC	
18	TFA	Pro-Flex® LP Align	OTTOBOCK 3R106-Pro
19	TFA	Pro-Flex® LP	OTTOBOCK 3R106-Pro
20	TTA	Pro-Flex® LP Align	
21	TTA	RUSH Foot HiPro	
22	TTA	RUSH Foot HiPro	
23	TTA	Pro-Flex® LP	
24	TTA	OTTOBOCK Triton 1C61	
25	TTA	Vari-Flex®	
26	TFA	OTTOBOCK Trias	OTTOBOCK 3F8O
27	TFA	Pro-Flex® Pivot	Rheo Knee® XC
28	TFA	Vari-Flex®	OTTOBOCK 3R60
29	TTA	Vari-Flex®	
30	TTA	Pro-Flex® Pivot	
31	TFA	Pro-Flex® LP	Rheo Knee® XC
32	TFA	Pro-Flex® LP Torsion	Rheo Knee® XC
33	TTA	Flex-Foot Assure®	
34	TFA	OTTOBOCK Meridium	OTTOBOCK X3
35	TFA	Flex-Foot Assure®	Total Knee® 2000
36	TTA	Pro-Flex® LP Torsion	
37	TFA	OTTOBOCK Taleo	OTTOBOCK C-leg
38	TTA	Pro-Flex® LP Align	
39	TTA	OTTOBOCK Meridium	
40	TTA	OTTOBOCK Taleo	
41	TTA	OTTOBOCK Triton 1C61	
42	TTA	Pro-Flex® XC	

TTA: participant with a unilateral transtibial amputation; TFA: participant with a unilateral transfemoral amputation

**Table 4:** Number of cases analysed per outcome variable

			Time	Speed	Heart rate	Oxygen consumption	VAS comfort	VAS fatigue	RPE
L-test	TTA	Current	*N* = 28	*N* = 28	*N* = 28	*N* = 28	*N* = 28	*N* = 28	*N* = 28
		TD	*N* = 28	*N* = 28	*N* = 28	*N* = 28	*N* = 28	*N* = 28	*N* = 28
	TFA	Current	*N* = 14	*N* = 14	*N* = 14	*N* = 14	*N* = 14	*N* = 14	*N* = 14
		TD	*N* = 14	*N* = 14	*N* = 14	*N* = 14	*N* = 14	*N* = 14	*N* = 14
Stair climbing	TTA	Current	/	*N* = 18	*N* = 18	*N* = 16	*N* = 18	*N* = 18	*N* = 18
		TD	/	*N* = 18	*N* = 18	*N* = 17	*N* = 18	*N* = 18	*N* = 18
	TFA	Current	/	/	/	/	/	/	/
		TD	/	/	/	/	/	/	/
Slope walking	TTA	Current	/	*N* = 12	*N* = 12	*N* = 12	*N* = 12	*N* = 12	*N* = 12
		TD	/	*N* = 12	*N* = 12	*N* = 12	*N* = 12	*N* = 12	*N* = 12
	TFA	Current	/	*N* = 25	*N* = 25	*N* = 24	*N* = 25	*N* = 25	*N* = 25
		TD	/	*N* = 25	*N* = 25	*N* = 24	*N* = 25	*N* = 25	*N* = 25
6MWT	TTA	Current	/	SS: *N* = 28 75% SS: *N* = 27 125% SS: *N* = 27	SS: *N* = 28 75% SS: *N* = 27 125% SS: *N* = 27	SS: * N* = 27 75% SS: *N* = 24 125% SS: *N* = 25	*N* = 28	*N* = 28	SS: *N* = 28 75% SS: *N* = 27 125% SS: *N* = 27
		TD	/	SS: *N* = 28 75% SS: *N* = 27 125% SS: *N* = 27	SS: *N* = 28 75% SS: *N* = 27 125% SS: *N* = 27	SS: *N* = 27 75% SS: *N* = 25 125% SS: *N* = 26	*N* = 28	*N* = 28	SS: *N* = 28 75% SS: *N* = 27 125% SS: *N* = 27
	TFA	Current	/	SS: *N* = 14 75% SS: *N* = 14 125% SS: *N* = 14	SS: *N* = 14 75% SS: *N* = 14 125% SS: *N* = 14	SS: *N* = 14 75% SS: *N* = 14 125% SS: *N* = 14	*N* = 14	*N* = 14	SS: *N* = 14 75% SS: *N* = 14 125% SS: *N* = 14
		TD	/	SS: *N* = 14 75% SS: *N* = 14 125% SS: *N* = 14	SS: *N* = 14 75% SS: *N* = 14 125% SS: *N* = 14	SS: *N* = 13 75% SS: *N* = 13 125% SS: *N* = 13	*N* = 14	*N* = 14	SS: *N* = 14 75% SS: *N* = 14 125% SS: *N* = 14
3mBWT	TTA	Current	*N* = 28	*N* = 28	*N* = 28	*N* = 28	*N* = 28	*N* = 28	*N* = 28
		TD	*N* = 28	*N* = 28	*N* = 28	*N* = 28	*N* = 28	*N* = 28	*N* = 28
	TFA	Current	*N* = 14	*N* = 14	*N* = 14	*N* = 14	*N* = 14	*N* = 14	*N* = 14
		TD	*N* = 14	*N* = 14	*N* = 14	*N* = 14	*N* = 14	*N* = 14	*N* = 14

Total sample size amounted 28 participants with a TTA and 14 participants with a TFA

VAS: visual analogue scale; RPE: rate of perceived exertion; Stair climbing: 2 min of stair climbing; Slope walking: 2 min of inclined treadmill walking at 10%; 3mBWT: 3-m Backward Walk test; 6MWT: 6 min of treadmill walking at 3 different speeds in consecutive blocks of 2 min; Current: participants’ current prosthesis; TD: Talaris Demonstrator; SS: self-selected speed; TTA: participant with a unilateral transtibial amputation; TFA: participant with a unilateral transfemoral amputation

## Abbreviations

TTA: Individual with a unilateral transtibial amputation
TFA: Individual with a unilateral transfemoral amputation
TD: Talaris Demonstrator
VAS: Visual analogue scale
RPE: Rating of perceived exertion
3mBWT: 3-m Backward Walk test
SS: Self-selected speed
VO_2_: Oxygen uptake
